# Chronic obstructive pulmonary disease as a risk factor for sarcopenia: A systematic review and meta-analysis

**DOI:** 10.1371/journal.pone.0300730

**Published:** 2024-04-18

**Authors:** Zhenjie Yu, Jingchun He, Yawen Chen, Ziqi Zhou, Lan Wang

**Affiliations:** 1 School of Nursing, Tianjin Medical University, Tianjin, China; 2 Department of Respiratory and Critical Care Medicine, Tianjin No. 4 Central Hospital, Tianjin, China; University of Turin: Universita degli Studi di Torino, ITALY

## Abstract

Sarcopenia prevalence and its risk factors in chronic obstructive pulmonary disease (COPD) vary partly due to definition criteria. This systematic review aimed to identify the prevalence and risk factors of sarcopenia in COPD patients. This review was registered in PROSPERO (CRD42022310750). Nine electronic databases were searched from inception to September 1^st^, 2022, and studies related to sarcopenia and COPD were identified. Study quality was assessed using a validated scale matched to study designs, and a meta-analysis was performed to evaluate sarcopenia prevalence. COPD patients with sarcopenia were compared to those without sarcopenia for BMI, smoking, and mMRC. The current meta-analysis included 15 studies, with a total of 7,583 patients. The overall sarcopenia prevalence was 29% [95% CI: 22%–37%], and the OR of sarcopenia in COPD patients was 1.51 (95% CI: 1.19–1.92). The meta-analysis and systematic review showed that mMRC (OR = 2.02, P = 0.04) and age (OR = 1.15, P = 0.004) were significant risk factors for sarcopenia in COPD patients. In contrast, no significant relationship was observed between sarcopenia and smoking and BMI. Nursing researchers should pay more attention to the symptomatic management of COPD and encourage patients to participate in daily activities in the early stages of the disease.

## 1. Introduction

Chronic obstructive pulmonary disease (COPD) is a condition characterized by chronic inflammation and extrapulmonary changes [1]. The extrapulmonary changes include malnutrition, changes in body composition, lower levels of physical activity and reductions in muscle mass or strength; these are common manifestations of COPD and have a negative impact on prognosis [2–4]. The presence of such factors is also closely related to sarcopenia, which is defined as a progressive and generalized skeletal muscle disorder characterized by the accelerated loss of muscle mass and function [5]. Sarcopenia has been estimated to occur in approximately 5–13% [6] of the ‘healthy’ older population. COPD patients may have an increased risk of developing sarcopenia [7]. However, due to the different management strategies for COPD in different countries, a considerable variation in prevalence is observed, ranging from 8.38% to 52.1% [8, 9]. In addition, the prevalence of sarcopenia in different studies also varied.

Sarcopenia negatively impacts health outcomes, and is an independent risk factor for mortality in COPD patients [10]. However, despite the association between sarcopenia and poor prognosis in COPD, the impact of sarcopenia on COPD patients cannot be accurately evaluated due to the variation in prevalence estimates. The variability may be attributed to several reasons. First, the definition of sarcopenia has not been standardized [11], with the presence of multiple organizations such as the definition of European Working Group on Sarcopenia in Older People (EWGSOP) or the Asian Working Group for Sarcopenia (AWGS). Second, inaccuracies in prevalence may arise from differences in the quality and quantity of the analyzed studies and the sampling strategies. Third, sarcopenia may have a different impact on COPD patients of different severity, leading to variations in prevalence estimates. Sarcopenia in COPD patients is highly prevalent during acute exacerbations but may improve after 6 months of recovery [12].

Although inflammatory factors and physical inactivity have been proved to be a significant correlation for sarcopenia, few researchers investigate the specific factors for sarcopenia in patients with COPD.

Furthermore, although some studies have explored the risk factors, the reported findings are often based on small sample sizes with conflicting results or may be limited to individual regions [13]. Investigating the potential risk factors in a systematic review can provide a theoretical basis for preventive practices, reduce bias, and increase persuasion.

To date, few systematic reviews have provided pooled estimates of sarcopenia. The available studies [6, 14] did not report the specific relationship between COPD and sarcopenia. This systematic review aimed to determine the pooled sarcopenia prevalence in COPD and identify the risk factors to provide evidence-based recommendations for sarcopenia. The results could improve awareness, control and treatment and promote better nursing management for sarcopenia in COPD.

## 2. Methods

This meta-analysis was registered in the International Prospective Register of Systematic Reviews (number CRD42022310750).

### 2.1. Search strategy

Articles published in English or Chinese were searched on PubMed, Cochrane Library, MEDLINE, Embase, Web of Science, China Knowledge Resource Integrated Database, Wanfang Database, Chinese Biomedical Database, and Weipu Database from inception to September 1^st^, 2022. The following search terms were used: ‘COPD’, ‘chronic obstructive pulmonary disease’, ‘sarcopenia’, ‘relative risk’, and ‘cohort studies’. All included studies were screened to ensure that all eligible studies were captured, and the reference lists of the identified articles were manually searched to further identify relevant publications. Gray literature was also searched. Some authors were contacted via e-mail to obtain details or for clarification. The study did not require the approval of an ethics committee since it is based entirely on previously published studies.

### 2.2. Study selection

After removing duplicate studies, two investigators independently assessed the eligible publications by screening titles and abstracts according to the inclusion and exclusion criteria. Full-text articles were retrieved when at least one reviewer decided that an abstract was eligible for inclusion. Each publication was assessed independently by both investigators for final study inclusion, and disagreements were resolved by discussion. The inclusion criteria were as follows: (1) all studies had to be case–control, longitudinal or cross-sectional designs; (2) assessment of sarcopenia had to be implemented according to the sarcopenia consensus criteria (muscle mass and muscle strength or physical performance are combined); (3) the prevalence of sarcopenia (primary outcome) in COPD patients had to be reported. The exclusion criteria were as follows: (1) studies contained incomplete data. (2) the language of the publication was other than English or Chinese.

### 2.3. Quality assessment

**Fig 1:**
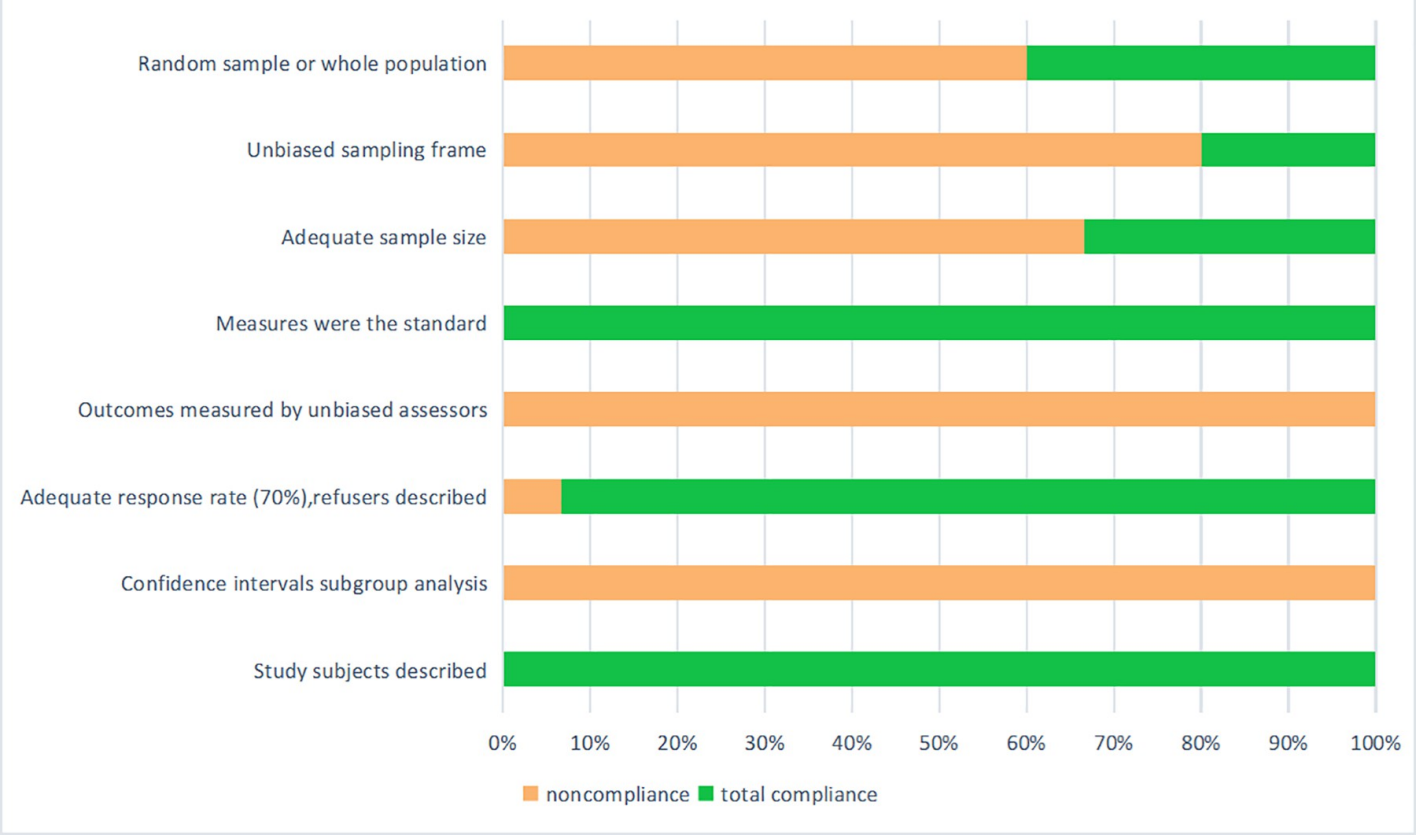
Critical appraisal of studies including the prevalence or incidence of sarcopenia in COPD patients.

The quality of the included studies was evaluated independently by two investigators with the tool for disease prevalence quality developed by Loney et al [15]. Any disagreement regarding the quality of studies was resolved by a senior investigator. The total score of the evaluation items was 8 points. Each item that was completely compliant was scored 1, and noncompliant or partly compliant items were scored 0. A higher cumulative score indicates a smaller risk of bias in the study, as shown in Fig 1.

### 2.4. Data extraction

The data were independently extracted by two researchers. The extracted data included the name of the first author, year of publication, study area, study type, sample size, mean age of patients, statistical model, other factors adjusted for in the model, measures, outcomes, and other subgroup analysis results.

### 2.5. Statistical analysis

The extracted data were analyzed by R.4.2.1 software package (meta) and Revman 5.4. Heterogeneity among studies was tested using Cochrane’s Q statistic. The degree of heterogeneity was assessed using the I^2^ statistic, with I^2^ values of 25%, 50%, and 75% indicating low, moderate, and high heterogeneity, respectively. Pooled prevalence and 95% confidence intervals (CIs) for sarcopenia were calculated using a random-effects model when Cochrane’s Q statistic detected significant heterogeneity; otherwise, a fixed-effects model was used. In this study, P < 0.05 was considered statistically significant. The findings were illustrated in the form of forest plots. Subsequently, the proportions of participants diagnosed with sarcopenia were extracted from all included studies to calculate the pooled prevalence. To assess the risk factors of sarcopenia in COPD, the odds ratios (ORs) and associated 95% CIs were extracted from included studies, and all eligible available data were summarized. A funnel plot was generated to evaluate publication bias, and the asymmetry was tested by using Egger’s linear regression method (p < 0.1 was considered significant).

In stratified meta-analyses, the literature data were divided into subgroups according to smoking history, mMRC, age, and BMI. Pooled estimates of sarcopenia prevalence with 95% CIs were then calculated.

## 3. Results

### 3.1. Study process

**Fig 2:**
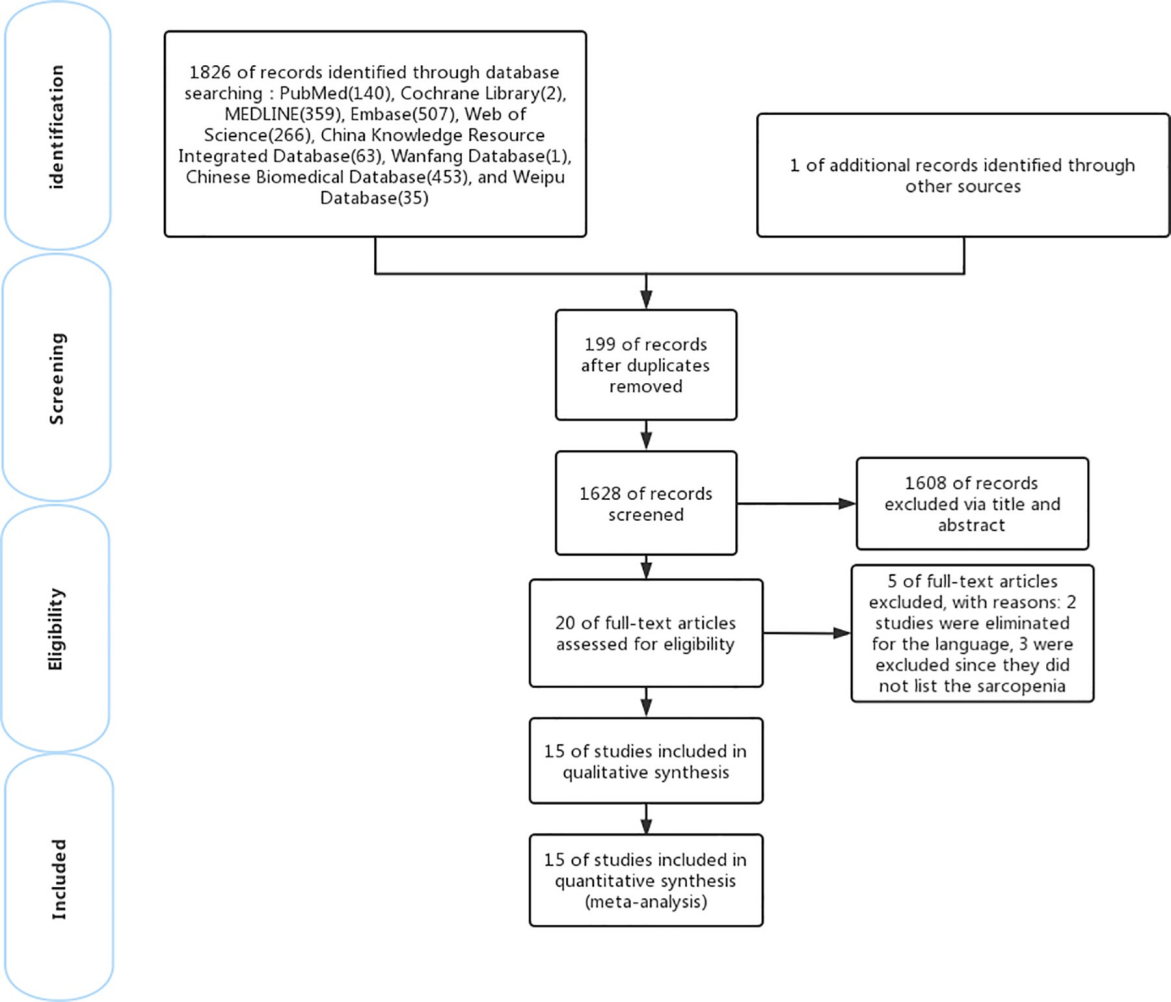
Preferred Reporting Items for Systematic Reviews and Meta-Analyses (PRISMA) flow diagram for the study selection process.

The initial search retrieved 1826 articles, of which 199 were duplicates. After screening titles and abstracts, 20 articles were selected according to the inclusion criteria and were evaluated in detail. Of them, 3 were excluded since they did not list the sarcopenia diagnostic criteria, and 2 studies were eliminated for the language. Ultimately, a total of 15 studies (2 in Chinese and 13 in English) met the inclusion criteria and were included in the meta-analysis (Fig 2).

### 3.2. Characteristics of the included studies

The characteristics of the 15 included studies are summarized in Table 1. These articles were published between 2015 and 2021, with three studies conducted in China, one in Russia, one in Brazil, one in Japan, one in Greece, one in Colombia, one in Mexico, one in Turkey, one in Korea, one in France, one in the UK, and two studies were multi-center studies. As evidenced by the Methodological scoring system (Fig 1). Ten of the studies [7–9, 11, 13, 16–20] were not prospective studies, and only five studies included [9, 17, 20–22] sample sizes over 300. Furthermore, only three studies used an unbiased sampling frame [7, 17, 19], two were multi-center clinical trials [7, 21], one was based on database [17]. None of the studies indicated whether the analysts were blinded, and none reported the 95% confidence intervals for the prevalence of sarcopenia. One study didn’t report the response rate [19].

**Table 1:** Characteristics of all included studies.

First author (year)	Study area	study types	Sample size	Sex(M:F)	Sample age	Statistical model	Outcomes	incidence	event	n	OR	95% CI	Subgroup	OR	95% CI
A. Suleymanova2019	Russia	Cross-sectional study	86	68:18	66.6±8.7		Questionnaire Muscle strength DXA	44.10%	38	62	2.8	1.1–6.8	mMRC ≥2 grades	4.6	1.80–11.60
Bruna Espíndola 2021	Brazil	cohort study	208	95:113	67.6± 10.1	Logistic regression	Handgrip strength, CC	16.30%	34	208			male	1.17	0.42–3.30
Maria Tsekoura 2020	Greece	Cross-sectional study	69	Not stated	71.33 ± 7.48	Logistic regression	HGS; BIA; SMM;Gait speed	24.60%	17	69	0.8	0.41–0.94	BMI	2.94	1.52–5.69
													Smoking	0.82	0.23–2.88
Carlos Andrés Celis2016	Bogota	Cross-sectional survey	2000	732:1268	>60	Logistic regression	Gait speed; Gait strength; Muscle mass	8.38%	28	334	1.9	1.1–3.3	Smoking	1.12	1.03–1.21
													age71-80	2.24	0.54–10.70
													age>80	10.07	1.77–57.2
													BMI	0.66	0.55–0.80
H. Nakamura2017	Japan	Cross-sectional study	61	57:4	73.7 ± 7.3		BIA	31.10%	19	61			BMI	0.65	
LIAN J2017	China	Case-control study	COPD 96 nCOPD 52	83:65	61±5.8	Logistic regression	Handgrip strength	28.13%	27	96			BMI	0.422	0. 29–0. 66
													age	1.223	1. 08–1. 39
													Smoking	38.93	3. 73–406. 88
													mMRC	1.065	0. 77–1. 47
Pavol Joppa MD2016	Germany\Belgium\ UK\ Omaha\ Boston\Kosice,	longitudinal, multicenter prospective study	2548 COPD: 2000	1589:959	COPD 63.50±7.10 NCOPD 54.80±9.00	Logistic regression	FFMI	24%	485	2000	1.5	1.1–2.1			
Panita Limpawattana2017	Southeast Asia	cross-sectional study	121	112:9	70.00±9.00	Logistic regression	SMM	24%	29	121			age65–74	2	0.40–10.80
													age > = 75	13.3	2.20–79.90
													mMRC	1.9	1.10–3.30
													BMI	0.04	0.003–0.60
Sarah E Jones2015	UK	Case-control study	622	354:268	73.00±8.00		SMM; SMI	14.50%	90	622					
NathalieMartínez-Luna2022	Mexico	cross-sectional study	185	102:83	72.20±8.39	linear regression model	ASMMI Handgrip strength	42%	78	185					
ZHANG Ji-you 2021	China	Cross-sectional study	3016	2512:504	65~84	Logistic regression	Mucle mass Pace measurement; Grip forece	27.49%	829	3016			BMI	0.898	0.87–0.93
													Smoking	1.574	1.41–1.76
Baiyang Lin 2021	China	Original study	73	59:14	73.21±9.54	Multiple regression analysis	Pace measurement; Grip forece measurement; DEXA	38.36%	28	73			age	1.11	1.01–1.23
L.Perrot 2020	French	observational study	54	37:17	68.00±9.00	multivariate mixed model	SMI HGS	48%	26	54					
Havva DEM2020	Turkey	cross-sectional study	219	196:23	66.90±10.10	regression analysis	Handgrip strength, BIA, Gait speed	52.10%	114	219			age50–59	1	
													age60-69	0.759	0.31–1.87
													age70-79	2.327	0.81–6.68
													age>80	2.611	0.66–10.38
													mMRC 0–1	1	
													mMRC ≥ 2	3.347	0.75–14.87
													BMI ≥ 30	1	
													BMI < 30	36.34	12.39–106.59
Y. K. Jeon2015	Korea	cross-sectional study	463	256:207	71.20±4.30	Logistic regression	DXA	20.50%	95	463			males	2.9	1.50–5.63
													females	9.15	1.53–54.77

DXA = Dual-emissionX-ray Absorptiometry; CC = Calf Circumference; HGS = Hand Grip Strength; BIA = Bioelectrical Impedance Analysis; SMM = Skeletal Muscle Mass; FFMI = Fat-free Mass Index; SMI = Skeletal Muscle Index; ASMI = Appendicular Skeletal Muscle Mass Index; DEXA = Dual Energy X-ray Absorptiometry

**Fig 3:**
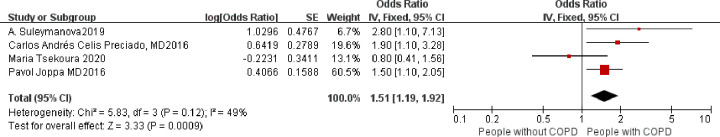
Odds ratios for COPD complicated by sarcopenia.

Four studies reported ORs for COPD complicated by sarcopenia. The OR between COPD and controls ranged from 0.8 to 2.8. The results of the meta-analysis of ORs are shown in Fig 3. Based on a random-effects model-based meta-analysis conducted on all data points, the overall OR of sarcopenia was 1.51 (95% CI: 1.18–1.92, I^2^ = 49%, P = 0.0008).

### 3.3. Prevalence of sarcopenia

**Fig 4:**
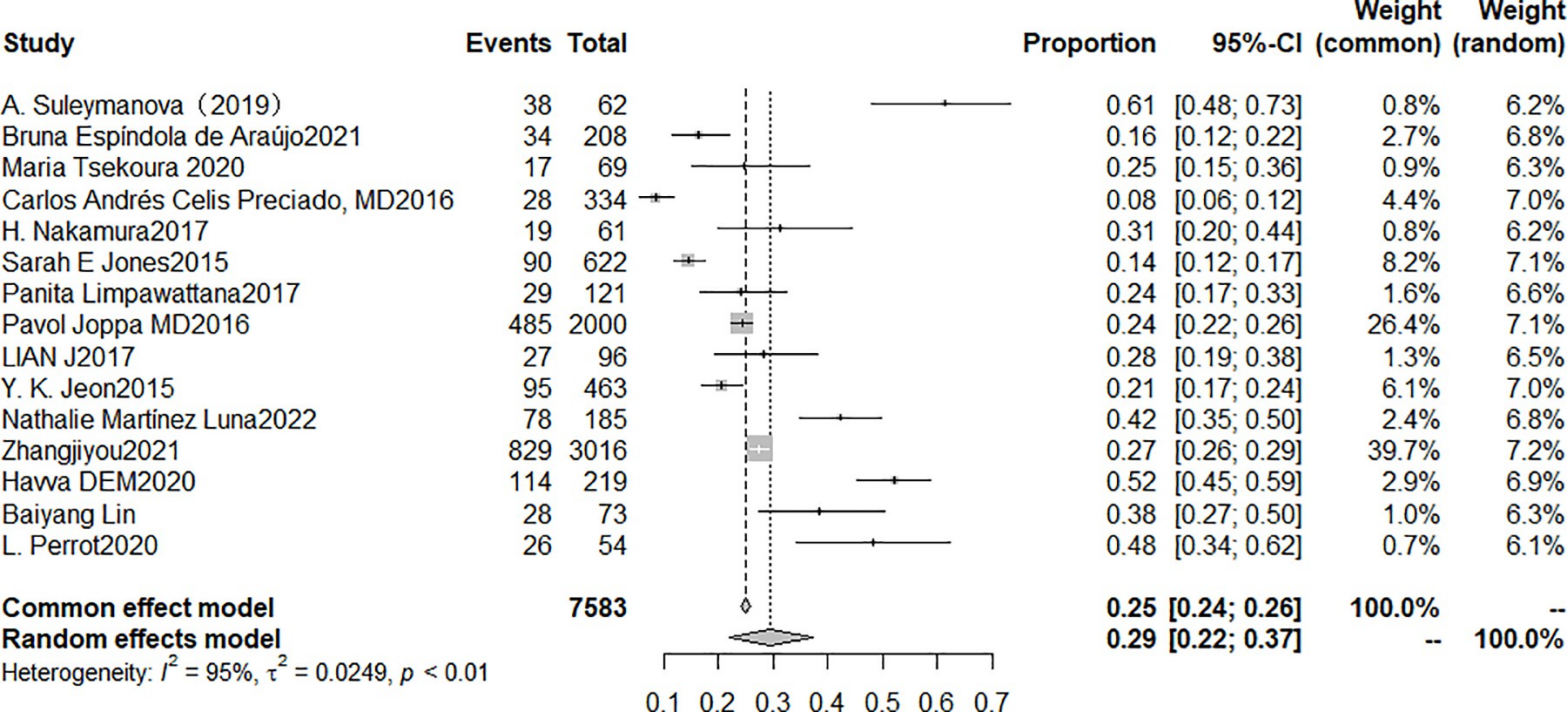
Forest plot of prevalence of sarcopenia in COPD.

In the 15 studies available for the meta-analysis, the prevalence of sarcopenia in COPD ranged from 8.38% to 52.1%. Based on a random-effects model-based meta-analysis conducted on all data points, the overall sarcopenia prevalence in COPD patients was estimated to be 29% (95% CI: 22% to 37%, I^2^ = 95%, P < 0.01), as shown in Fig 4.

**Fig 5:**
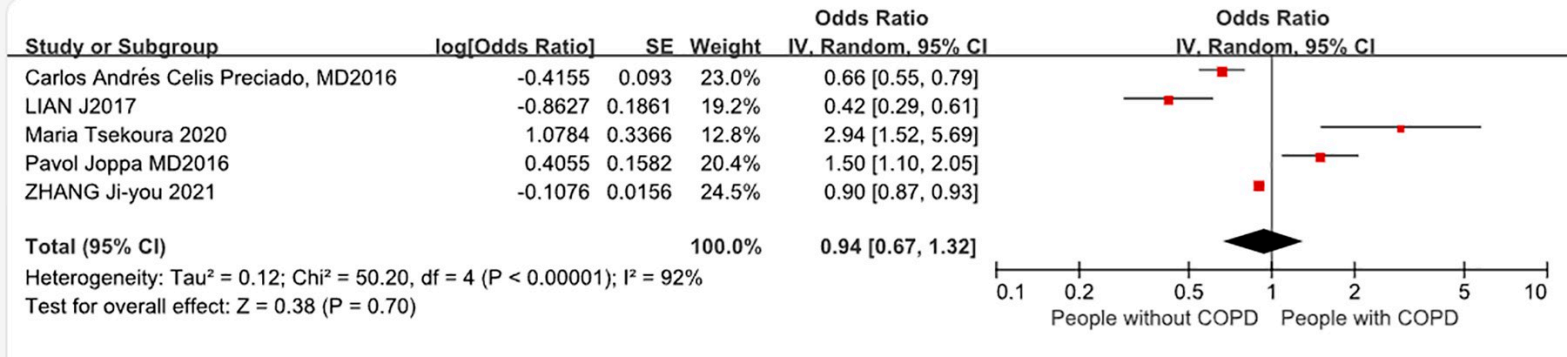
Forest plot of body mass index.

**Fig 6:**
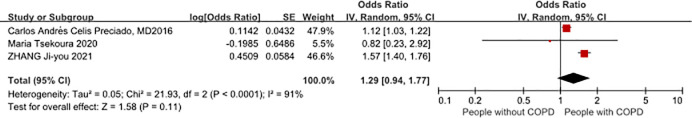
Forest plot of smoking.

**Fig 7:**
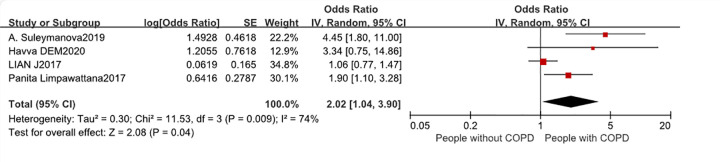
Forest plot of mMRC.

**Fig 8:**
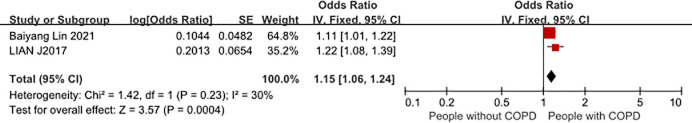
Forest plot of age.

Most of the included studies contained the following four influencing factors: BMI, smoking, age, and mMRC. Some studies were excluded from the risk factor meta-analysis due to skewed confidence intervals [8, 16, 23]. In addition, three other studies [8, 16, 23] related to age were not included in the age meta-analysis due to the different subgroups in each study. Therefore, the meta-analysis compared sarcopenia in COPD patients based on BMI, smoking, mMRC and age (Figs 5–8). After adjusting for confounding factors, mMRC (OR:2.02[1.04–3.93], P = 0.04) and age (OR:1.15[1.06–1.24], P = 0.004) were associated with a significantly higher risk of developing sarcopenia in COPD patients. In contrast, no statistical significance was observed with BMI (P = 0.47) and smoking(P = 0.06).

### 3.4. Publication bias

**Fig 9:**
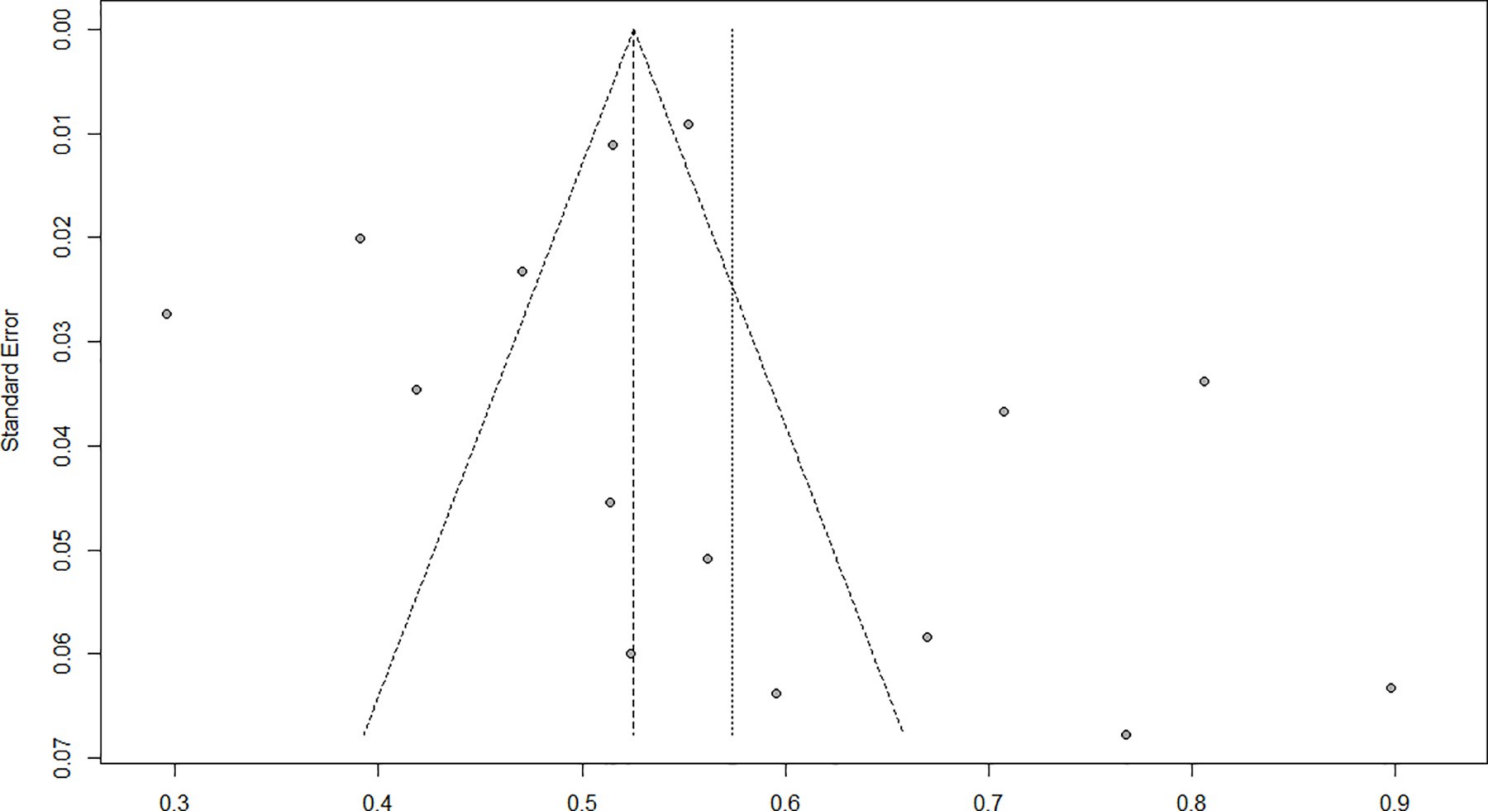
Funnel plot.

Funnel plot symmetry (Fig 9) indicated no publication bias in between-study heterogeneity. The results of Egger’s weighted regression test further confirmed the funnel plot symmetry (P = 0.467).

## 4. Discussion

In this comprehensive systematic review and meta-analysis encompassing 15 studies with a total of 7,583 patients diagnosed with COPD, we observed a sarcopenia prevalence rate of 29%. The data further indicates that individuals with COPD were 1.51 times more likely to develop sarcopenia in contrast to those without COPD. It’s pivotal to highlight that significant variations in the incidence of sarcopenia were associated with mMRC scores and the age of the participants, whereas factors like BMI and smoking patterns showed no notable correlation.

Fig 3 delineates that among the surveyed populations, the Turkish cohort exhibited the highest incidence of sarcopenia, whereas the Bogota group reflected the lowest [8, 9]. Several underlying determinants can elucidate the disparities in reported prevalences: 1. Sample size differences: the sample size of the included studies ranged from 54 subjects in a study conducted in France [12] to 3016 subjects in a Chinese Study [20]. 2. Inconsistency in the sex ratio of subjects: male subjects made up the majority of the study population in different studies. 3. Use of different diagnostic methods, including pace measurement, handgrip strength, questionnaire, calf circumference; bioelectrical impedance analysis; skeletal muscle mass; fat-free mass index; skeletal muscle index; appendicular skeletal muscle mass index and dual-energy x-ray absorptiometry.

Individuals diagnosed with COPD exhibit a markedly increased susceptibility to sarcopenia, a finding consistent with prior studies [24]. Muscle mass is an important predictor of survival in COPD patients. A confluence of elements such as diminished appetite, restricted mobility, and glucocorticoid administration can catalyze muscular deterioration [25, 26]. In conjunction with relevant studies, the influence factors included chronic inflammatory responses, oxidative stress, insulin resistance, inadequate protein intake or excessive catabolism, and decreased physical activity [27]. Lin [28] also showed that people with COPD had significantly higher concentrations of the inflammatory factors IL-6 and IL-10, leading to increased degradation of muscle proteins and decreased muscle tissue, which exacerbated sarcopenia [29]. Age-related muscular changes yield an imbalanced mitochondrial equilibrium, amplifying oxidative stress. This imbalance, in turn, impedes protein accretion while promoting its breakdown, ushering in sarcopenia [30]. Concurrently, an inverse relationship exists between insulin resistance and muscular volume. In summation, a myriad of interrelated factors accentuates the likelihood of nutritional deficits and muscle wasting, ultimately manifesting as sarcopenia.

The discrepancy in sarcopenia incidence between genders remains under-investigated, with a solitary study making gender-based comparisons, thus rendering it ineligible for inclusion in the meta-analysis. One plausible explanation centers on females’ heightened susceptibility to affective disorders such as anxiety and depression, which can precipitate elevated cortisol concentrations. Augmented cortisol levels can catalyze enhanced protein catabolism within muscular tissues, culminating in diminished muscle volume. Excessive fructose consumption has been implicated in the onset of insulin resistance, a condition that potentiates protein degradation while stifling protein synthesis, thereby further eroding muscle mass.

The discrepancy in sarcopenia incidence between genders remains under-investigated, with only one solitary study making gender-based comparisons, thus rendering it ineligible for inclusion in the meta-analysis [17]. However, preliminary indications suggest that females may exhibit a heightened incidence relative to their male counterparts. One plausible explanation centers on females’ heightened susceptibility to affective disorders such as anxiety and depression, which can precipitate elevated cortisol concentrations [31]. Augmented cortisol levels can catalyze enhanced protein catabolism within muscular tissues, culminating in diminished muscle volume [32]. Secondly, dietary habits reveal that females display a predilection for snack consumption, particularly those laden with high fructose syrup [33]. Excessive fructose consumption has been implicated in the onset of insulin resistance, a condition that potentiates protein degradation while stifling protein synthesis, thereby further eroding muscle mass [34].

The mMRC scale serves as a tool for gauging the intensity of dyspnea experienced by COPD patients, wherein escalated scores denote exacerbated dyspneic manifestations [35]. Our investigative endeavors have discerned a heightened predisposition towards sarcopenia amongst individuals registering elevated mMRC scores, a correlation corroborated by antecedent research [36, 37]. The severity of dyspnoea correlates with the ability to perform physical activity, and lack of exercise is a major risk factor for sarcopenia. Regular physical exertion augments mitochondrial proliferation, enzymatic vigor, and amplifies the transcription of insulin-like growth factor (IGF), all of which culminate in an uptick in muscle protein anabolism and the enhancement of muscular vitality [38]. Conversely, diminished outdoor pursuits can precipitate inadequate solar exposure, potentially ushering in a deficit of vitamin D—a micronutrient independently tied to muscular atrophy and debility [38]. In addition, a higher mMRC score represents a poorer pulmonary function, which was also associated with sarcopenia in this study [39].

The present study showed a higher occurrence of sarcopenia with age, which was consistent with the review in Lancet [5] and cross-sectional studies in South Asia and Bogotá [9, 36]. However, a study [8, 17] conducted in Turkey [8] showed controversial results. This may be related to different countries having different awareness of healthy aging [36]. In developed countries, there’s a pronounced emphasis on health literacy, prompting individuals to conscientiously incorporate physical activity into their routines to stave off the ravages of age-induced physiological degradation and muscular attrition. While a duo of Chinese studies integrated into our meta-analysis endorsed the notion that senescence exacerbates the susceptibility to sarcopenia, they remained non-committal about the specific age demarcations. It’s imperative for ensuing inquiries to stratify age categories in alignment with the stipulations laid out by the WHO, ensuring the provision of granular data conducive to meticulous intervention strategizing.

The present meta-analysis demonstrated no variation in the pooled prevalence of sarcopenia with the degree of BMI. Some studies [13, 40] showed that low BMI is associated with a higher risk of sarcopenia. This may be related to the fact that COPD is a chronic disease often complicated by malnutrition and hypoproteinemia, leading to sarcopenia [41]. However, other studies [7, 8] showed that COPD patients with sarcopenia might have a higher BMI. Patients with COPD may exhibit a considerable loss in lean body mass (LBM) but store extra fat, resulting in a high BMI [42]. The Fat-Free Mass Index (Ffmi) is more suitable for assessing patients with COPD compared to healthy people due to long-term hormone use, leading to central obesity, which masks the presence of sarcopenia [43]. All these factors point to the need for care for all patients with COPD, not only patients with low BMI. Meanwhile, it is worth noting that because the original study did not stratify gender, this study was unable to explore the actual effect of sex-specific BMI on sarcopenia. However, BMI varies by gender, so it is recommended that future studies be explored in depth for patients of different genders.

While smoking is an acknowledged risk factor for COPD, it is also implicated in skeletal muscle impairment, potentially through intertwined mechanisms that underpin both COPD and sarcopenia [44]. Carbon monoxide, a prominent constituent of cigarette smoke, compromises the hemoglobin’s oxygen-carrying capacity and may further impede intracellular oxygen diffusion. These disruptions can impair mitochondrial respiratory processes, diminishing ATP synthesis, and subsequently impinging upon skeletal muscle functionality [24]. The results of this study suggest that smoking is not a risk factor for sarcopenia in patients with COPD. This may be related to the large proportion of patients who quit smoking in the original data (82.4%) [7]. Smoking cessation can reverse the loss of muscle mass in the extremities and ameliorate diaphragm atrophy [45]. Meanwhile, we can’t rule out bias in the understanding of non-smoking in patients in the original study. For example, a response of "no" to smoking might convey different meanings for men and women: for men, it might indicate they have quit smoking, whereas for women, it could imply they never smoked. In short, gender might be a complicated confounder influencing both the outcome and the variables. As a forward-looking recommendation, ensuing studies should encompass more expansive sample sizes and ensure a balanced representation of active smokers, former smokers, and non-smokers across both male and female categories.

The foremost advantage of our analysis is anchored in its rigorous methodological approach, encompassing an exhaustive literature review across nine distinct databases and spanning both English and Chinese academic contributions. Such an extensive survey fortified the precision of data pertaining to sarcopenia within the COPD context and facilitated nuanced stratifications, taking into account parameters like mMRC, gender, smoking habits, and BMI. To the best of our comprehension, this investigation stands unparalleled, presenting both a quantification of sarcopenia’s prevalence in COPD patients and a meticulous evaluation of correlated risk determinants. Yet, there were inherent constraints to our study. Firstly, the constituent studies adopted a range of methodologies for sarcopenia detection. However, all original studies used objective indicators to ensure the accuracy of the studies. Secondly, the systematic review did not include unpublished articles and studies that did not use valid and objective assessment methods, likely contributing to publication bias. However, we searched 9 major databases to ensurethe original articles as comprehensive as possible. Lastly, the languages of the included studies were limited to English and Chinese, the exclusion of works published in other languages limited the comprehensiveness of the included literature. However, our study has included more than 10 countries, including the United Kingdom, France, and China. The results are highly reliable. Bearing in mind these limitations, there’s a compelling case for future scholarly endeavors to delve deeper, furnishing more holistic perspectives on strategies for thwarting sarcopenia’s onset within the COPD populace.

## 5. Conclusion

In conclusion, this study revealed a pooled prevalence of sarcopenia in COPD of 29%. Such a high prevalence indicates the need for raising public awareness of sarcopenia in COPD. Meanwhile, the study showed that dyspnea and age constituted constituted risk factors for sarcopenia in COPD. The researchers should do early and regular screening, the whole process of managing high-risk groups, and formulating targeted measures according to risk factors for precise prevention.

## Supporting information

S1 Checklist PRISMA 2020 checklist. (DOCX)

## List of abbreviations

COPD: Chronic obstructive pulmonary disease
OR: odds ratio
CI: confidence interval
EWGSOP: European Working Group on Sarcopenia in Older People
AWGS: Asian Working Group for Sarcopenia
LBM: lean body mass
DXA: Dual-emissionX-ray Absorptiometry
CC: Calf Circumference
HGS: Hand Grip Strength
BIA: Bioelectrical Impedance Analysis
SMM: Skeletal Muscle Mass
FFMI: Fat-free Mass Index
SMI: Skeletal Muscle Index
ASMI: Appendicular Skeletal Muscle Mass Index
DEXA: Dual Energy X-ray Absorptiometry
